# A descriptive analysis of depression and pain complaints among patients with cancer in a low income country

**DOI:** 10.1371/journal.pone.0193713

**Published:** 2018-03-07

**Authors:** Melkam Alemayehu, Negussie Deyessa, Girmay Medihin, Abebaw Fekadu

**Affiliations:** 1 Addis Ababa University, College of Health Sciences, School of Medicine, Department of Psychiatry, Addis Ababa, Ethiopia; 2 Addis Ababa University, College of Health Sciences, School of Public Health, Addis Ababa, Ethiopia; 3 Addis Ababa University, Aklilu Lemma Institute of pathobiology, Addis Ababa, Ethiopia; 4 Center for Innovative Drug Development and Therapeutic Trials for Africa (CDT-Africa), Addis Ababa University, College of Health Sciences, Addis Ababa, Ethiopia; 5 Department of Global Health and Infection, Brighton and Sussex Medical School, University of Sussex, Brighton, United Kingdom; 6 King’s College London, Institute of Psychiatry, Department of Psychological Medicine, Centre for Affective Disorders, London, United Kingdom; Chiba Daigaku, JAPAN

## Abstract

**Background:**

In high income countries, cancer is one of the leading causes of death, with co-morbid depression contributing to the risk of increased mortality. However, both cancer and depression are neglected conditions in low income countries. The current study assessed the magnitude of depression and the association of pain complaints with depression among patients with cancer in a low income country.

**Method:**

In this cross-sectional study participants were 390 patients with established diagnosis of cancer, who were recruited consecutively when visiting a tertiary treatment centre in Addis Ababa, Ethiopia. The occurrence of depression was determined using the nine items Patient Health Questionnaire (PHQ-9). Major depressive disorder was confirmed: (1) when five or more of the PHQ-9 symptoms were endorsed as occurring for at least ‘more than seven days’, with the exception of suicidal ideation item which counted as a positive rating if it had occurred even once in the previous fifteen days. (2) one of the symptoms has to be either depressed mood or loss of interest. Pain complaint was measured by Numeral Rating Scale (NRS) and severity of pain was assessed using Verbal Rating Scale (VRS).

**Results:**

The prevalence of major depressive disorder was 16.4% (95%CI: 13.1%, 20.4%), and subthreshold depression was 17.4% (95%CI: 14.0%, 21.5%). Pain complaints occurred in 69.0% (95%CI: 64.3%, 73.4%) of the participants. The odds of having a major depressive symptom was over four times higher among participants who had pain.

**Limitations:**

The study was cross sectional and liable to recall bias. Recruitment was carried out in a tertiary referral hospital, which might lead to the selection of more economically well-off and educated participants limiting generalizability of the study. Moreover, we did not control for cancer types, which may be related to pain and the experience of depression. Some of the somatic symptoms in PHQ9 may also be related to the cancer itself.

**Conclusions:**

This study highlights the clinical significance of both depression and pain complaints in patients with cancer in a low income country. Exploration of the impact of depressive disorders on quality of life and outcome of cancer is an important area for further research in low income countries.

## Introduction

One in six deaths worldwide are due to cancer, establishing it as the second leading cause of death globally [1]. Approximately 14 million new cases of cancer were expected in 2012, with anticipated increase of 70% over the coming 20 years [2]. More than 70% of all deaths occur in low and middle-income countries[3, 4]. In Africa, the incidence of cancer and associated mortality continues to rise [5] with 645,000 new cases and 456,000 deaths in 2012 [6]. However, it receives little attention from a public health perspective. Similarly, although cancer is one of the major causes of death in Ethiopia, it is a far more neglected problem[7]. Despite the limited data on the epidemiology of cancer in Ethiopia, in 2008, the number of new cases of cancer was reported to be 51,700, with 41,600 deaths. The probability of dying from cancer before the age of 75 was 9.4% [8]. Among non communicable diseases, cancer is responsible for the second highest mortality in the country next to cardiovascular diseases [9–11]. The five year prevalence of cancer was reported to be 0.22%. Breast cancer, cervical cancer and cancer of the esophagus were the most prevalent forms of cancer. Virtually no systematic data exists on the cost of cancer from Ethiopia. There is limited service for patients with cancer in Ethiopia. Although most referral hospitals provide diagnostic and limited treatment services, most of the care for cancer is provided by Tikur Anbessa Specialized Hospital (TASH), a tertiary referral hospital located in the capital city Addis Ababa. TASH has the only radiotherapy and palliative care service in the country [12].

Among other causes, one of the factors that seem to increase death related to cancer is depression [13, 14], one study for example demonstrating a 19% increase in the relative risk for mortality among the depressed group [13]. Early diagnosis and treatment of co-morbid depression, which affects 10.8% to 61.6% of patients with cancer [15, 16], may help to minimize expenditures for medical costs, symptom burden, poor self care practice and adherence, which are mechanisms by which depression may increase morbidity and mortality in chronic conditions [13, 17–20].

Depression is a common and most burdensome neuropsychiatric condition, with the Disability adjusted life years projected to rank second by 2020. From African regions, north Africa has the highest depression rates [21]. The latest study in Ethiopia that assessed about 10,000 adults reported the prevalence of depression to be 9.1% (95% CI: 8.39, 9.90) [22]. However, subthreshold conditions, i.e., occurrence of clinically significant symptoms of depression without meeting the criteria for major depressive disorder, are more common and may accompany physical illnesses. Patients with subthreshold states have a higher risk of developing a major depressive disorder in the long run, with an estimated risk of up to 67%. This risk of developing major depressive disorder increases when the severity of the depressive symptoms is higher and when co-morbid medical conditions are present. When these occur, suicidal ideation may arise in up to 90% [23]

Depression commonly co-exists with pain. Pain is the most important symptom affecting quality of life of patients. Pain is also a common problem in patients with cancer with reported prevalence of 44% to 87% [24, 25].

Pain and depression together can worsen the clinical condition of a patient than either condition alone; and there is strong association between the two [26]. Pain is a commonly reported complaint among patients with depression. For example, in a systematic review synthesizing 14 facility based studies, the mean prevalence of pain in depressed individuals was 65% [27].Depression may cause pain and pain may cause depression [27–29]; and in fact some have argued that pain might play a causal role in producing depression [26] and that both depression and pain may share the same neurotransmitter pathways [27]. Depression in patients with pain is associated with more pain and greater impairment. Treatment of both conditions at the same time has the potential of improving both conditions [27].Even though depression is a major public health problem everywhere and of importance in the course of cancer, few studies have explored its effect on patients with cancer either alone or as co-morbid condition with pain in low income countries like Ethiopia. Therefore, this study assessed the magnitude of depression and pain complaints and their associations as well as the level of functional impairment due to depressive symptoms were evaluated among adult patients with established diagnosis of cancer attending a tertiary treatment center in Addis Ababa, Ethiopia.

## Materials and methods

### Study design and setting

This cross-sectional study was conducted in a public tertiary treatment centre in Addis Ababa. In addition to inpatient services, the centre provides radiotherapy and chemotherapy services. The Radiotherapy unit accepts referrals from all over the country and offers care for an average of 118 patients per week. Women make up 70% of the patients using this service.

### Study participants

Study was conducted in January 2012. Patients, 18 years of age and above, with established diagnosis of cancer for a minimum of one month were eligible for the study. Patients who were unable to communicate either because of illness or inability to speak the language of interview (Amharic) were not included. Inpatients were approached in the wards while outpatients were approached while waiting for their follow up or therapy. Interviews were carried out in a private room near the waiting area. Consenting consecutive patients were recruited until the required sample size (shown below) was obtained. The interview was conducted in Amharic and took an average of 20 minutes.

#### Sample size

Sample size was calculated assuming that the prevalence of depression and pain among patients with cancer to be 37% and 73% respectively [25] with an additional assumption of non-response rate of 10%, 95% level of confidence and a margin of error of 5%.

### Instrument and data collection procedures

Depression was assessed using a validated Amharic version of the 9-item of the patient health questionnaire (PHQ-9) as the criterion standard for assessing both major depression and Subthreshold depression [30, 31]. The PHQ rates for each of the 9 symptoms of depression contained in the Diagnostic and Statistical Manual of Mental Disorders, 4^th^ revision (DSM-IV) through patients’ self-report [32]. Each symptom is rated over a 2-week period and in four severity categories based on duration and persistence: “0” (not at all), “1” (symptoms occurred for less than seven days), “2” (symptoms occurred for more than seven days), or “3” (symptoms occurred nearly every day for at least the two week period). The PHQ-9 has demonstrated acceptable reliability, validity, sensitivity, and specificity [30]. The instrument was also used to assess depression co-morbidity with chronic illness including cancer [33] and was previously validated in Ethiopia [31].As briefed in Table 1 diagnosis of major depressive disorder was made if five or more symptoms have persisted for over 50% of the time and sub threshold depression was identified if among the 9 items of the PHQ 9, two to four of the symptoms have been persisting for more than 50% of the time for all symptoms. In either of these subtypes, suicidal ideation item is an exception which is counted as a positive rating if it had occurred even once in previous fifteen days. For the score based classification, we used the PHQ score range of 5–9 for mild, 10–14 for moderate, 15–19 for moderately severe, and above a score of 20 for severe depression. However, since several studies have used a binary categorization based on a cutoff value of 10 as the threshold for diagnosing depression, we have conducted a sensitivity analysis with this cutoff.

**Table 1:** Depression diagnosis: Criterion based and score based classification.

Basis for classification	Class or condition	Symptom or score specification
**Criterion-based**		
	**Major depression**	1 or 2 DSM Core symptoms
		At least 3 additional symptoms (giving a total of 5 or more symptoms)
	**Sub-threshold depression**	1 or 2 DSM Core symptoms
		0 to 3 additional symptoms (giving a total of 2–4 symptoms)
**Score-based**		
	**Mild**	5–9
	**Moderate**	10–14
	**Moderately severe**	15–19
	**Severe**	20 and higher

Functional impairment of the patient as the result of depressive symptoms was assessed using the 10^th^PHQ item with four response categories. The administered item states “If you checked off any problems, how difficult have these problems made it for you to do your work, take care of things at home, or get along with other people?” and the response categories are “Not difficult at all”, “Somewhat difficult”, “Very difficult” and “Extremely difficult”.

Pain was assessed using patients’ self report in Numeric and Verbal Rating Scale (NRS),which measures perceived pain on a 0–10 scale (0 = no pain and 10 = worst imaginable pain). Pain ratings of more than three usually indicate pain that interferes with daily activities and pain rating of 10 is the most severe pain imaginable. Verbal Rating Scale (VRS) is an ordinal scale of measurement for rating pain complaint based on the patient’s description. The response categories of VRS are none, mild, moderate, or severe. Both NRS and VRS are a validated tests in measuring pain exacerbation and have been used for measuring pain extent on cancer patients among the two measures NRS is a preferred option [24, 34–36]. However, in Ethiopian context VRS has been the most common approach to assess pain[37, 38]

Additionally, Patients were directly interviewed using a local questionnaire consisting of the socio demographic characteristics, such as sex, age, educational status, marital status, residency, employment status, regular source of income and perceived social support. Clinical characteristics of patients were acquired by directly asking patients and using the medical records of the hospital. Patients were directly asked for their Belief on cancer curability, health satisfaction, presence of other chronic illness and presence and extent of perceived pain. Patients who had just one visit, were considered as a new patient, while patients who had more than one visit were considered as a follow up patient. Cancer type and stage, medication type and duration since diagnosis, were extracted from the medical records following the latest clinical visit. The diagnosis confirmed by the oncologist based on the histo-pathological and radiographic findings was used for the diagnostic information.

### Data management

Data were entered and cleaned using EpiInfo Version 3.5.4., and exported to the Statistical Packages for Social Sciences, version 20 (SPSS 20), for analysis. Descriptive statistical methods were used to summarize data on socio-demographic and clinical characteristics. Bivariate analysis was employed to determine unadjusted associations of socio-demographic and clinical characteristics, including pain, with depression. Factors found to be significant in bivariate analysis were included in multivariable logistic regression model to evaluate adjusted effects of different factors on the odds of having Major depression. The effect of pain on the odds of having depression was investigated in detail by adjusting for one variable at a time and then adjusting fully for potential confounding variables. The impacts of the severity of pain assessed with the two measures were adjusted separately in the multivariable logistic regression to avoid Multi-colinearity. However, the effect of all other variables was adjusted for the effect of pain as measured by VRS as VRS is more explicit.

### Ethical considerations

Ethical clearance was obtained from Haramaya University ethical clearance committee and the Institutional Ethical Review Board of the College of Health Sciences, Addis Ababa University (ref no 041/2011). Informed consent was obtained from all participants involved in the study. All participants with moderate or severe depressive disorder and/ or sucidality were referred to a nearby psychiatry clinic or the psychiatric hospital in Addis Ababa for a psychiatric assessment and management.

## Result

### Socio-demographic characteristics

Background characteristics of the study participants are summarized in Table 2. From the total of 395 adult cancer patients targeted for the study, 390 patients were enrolled and participated in the study, resulting in a response rate of 98.7%. Four patients did not consent and one did not finish the interview because of health issues. The respondents were predominantly female (73.1%; n = 285), with a mean age of 43.5 (SD = 13.0) years.

**Table 2:** Socio-demographic characteristics and correlates of depression among study participants (n = 390).

Socio-demographic characteristics of participants	Number (%) interviewed	Major Depression		Crude OR (95% CI)
		Number (%) Yes	Number (%) No	
Sex				
Female	285 (73.1)	45(15.8)	240(84.2)	0.58(0.47, 1.53)
Male	105 (26.9)	19(18.1)	86(81.9)	1.00
Age				
< 35	93 (23.8)	12(12.9)	81(87.1)	0.96(0.41, 2.27)
35–44	109 (27.9)	25(22.9)	84(77.1)	1.93(0.91, 4.11)
45–54	98 (25.1)	15(15.3)	83(84.7)	1.18(0.52, 2.67)
>54	90 (23.1)	12(13.3)	78(86.7)	1.00
Educational Status				
No formal education	111 (28.5)	18(16.2)	93(83.8)	1.62(0.67, 3.95)
Primary education(Grade 1–8)	84 (21.5)	19(22.6)	65(77.4)	2.45 (1.00, 5.98)
Secondary Education(Grade 9–12)	120 (30.8)	19(15.8)	101(84.2)	1.58(0.65, 3.81)
Higher education (Anything above grade 12 e.g. Diploma, Degree. . .)	75 (19.2)	8(10.7)	67(89.3)	1.00
Marital Status				
Single	61 (15.6)	9(14.8)	52(85.2)	1.00
Married	239 (61.3)	36(15.1)	203(84.9)	0.98 (0.44, 2.15)
Formerly Married	90 (23.1)	19(21.1)	71(78.9)	1.51(0.81, 2.80)
Residence				
Addis Ababa	213(54.6)	30(14.1)	183(85.9)	1.00
Outside Addis Ababa	177 (45.4)	34(19.2)	143(80.8)	1.45 (0.85, 2.48)
Employment status				
Unemployed	212 (54.4)	40(18.9)	172(81.1)	1.49 (0.86, 2.59)
Employed	178 (45.6)	24(13.5)	154(86.5)	1.00
Regular source of Income				
Yes	149 (38.2)	16(10.7)	133(89.3)	1.00
No	241 (61.8)	48(19.9)	193(80.1)	2.07 (1.13, 3.80)
Perceived Social support				
Good	319 (81.8)	44(13.8)	275(86.2)	1.00
Poor	71 (18.2)	20(28.2)	51(71.8)	2.45 (1.34, 4.50)

### Clinical characteristics of respondents

Clinical characteristics of study participants are summarized in Table 3. Participants were predominantly affected by Breast cancer (29.5%; 114/387) and cervical cancer (22.7%; 88/387). Sizable proportion of the respondents were in a post operational stage (43.6%) and on follow up (71.8%); more than half (51.6%) were taking chemotherapy. The majority had their diagnosis confirmed less than a year ago (64.4%) and (47.2%) reported to have symptoms of cancer for 1–3 years. More chronic illness was reported by 20.8%.

**Table 3:** Unadjusted effects of selected clinical indicators on the odds of having major depression.

Clinical Characteristics of participants	Number (%) interviewed	Major Depression		Crude OR (95% CI)
		Number (%) yes	Number (%) no	
Patient category				
New	110 (28.2)	12(10.9)	98(89.1)	1.00
Follow up	280 (71.8)	52(18.6)	228(81.4)	1.86(0.95, 3.64)
Cancer Type				
Breast	114 (29.5)	14(12.3)	100(87.7)	0.65 (0.29, 1.42)
Cervical	88 (22.7)	13(14.8)	75 (885.2)	0.52 (0.24, 1.12)
Head and Neck	38 (9.8)	5(13.2)	33(86.8)	0.56 (0.19, 1.65)
Colorectal	33 (8.5)	7(21.2)	26(78.8)	1.00 (0.38, 2.68)
Sarcoma	29 (7.5)	6 (20.7)	23(979.3)	0.97(0.34, 2.74)
Others	85 (22.0)	18 (21.2)	67(78.8)	1.00
Stage of cancer (n = 362)				
Early (I&II)	63 (17.4)	9 (14.3)	54 (85.7)	1.00
Advanced (III, IV &V)	141 (39.0)	30 (21.3)	111(78.7)	1.62 (0.72, 3.66)
Post Operational Stage	158 (43.6)	19 (12.0)	139 (88.0)	0.82 (0.35, 1.93)
Medication for cancer				
Radiotherapy	113 (29.3)	26 (23.0)	87 (77.0)	1.68 (0.94, 3.02)
Radiotherapy +chemotherapy	74 (19.2)	7 (9.5)	67 (90.5)	0.59 (0.25, 1.40)
Chemotherapy	199 (51.6)	30 (15.1)	169 (84.9)	1.00
Duration since case is confirmed				
≤ a year	251 (64.4)	39 (15.5)	212 (84.5)	1.00
1–3 years	125 (32.1)	24 (19.2)	101 (80.8)	1.29 (0.74, 2.26)
≥ 3 Years	14 (3.6)	1 (7.1)	13 (92.9)	0.42 (0.05, 3.29)
Belief that Cancer can be Cured				
Yes	227 (58.4)	24 (10.6)	40 (24.7)	1.00
No	162 (41.6)	40 (24.7)	122(75.3)	2.77 (1.59, 4.82)
Health Satisfaction				
Poor/Fair	166 (42.6)	48 (21.4)	176 (78.6)	2.56 (1.39, 4.69)
Good	224 (57.4)	16 (9.6)	150 (90.4)	1.00
Presence of Other chronic Illness				
Yes	81 (20.8)	19 (23.5)	62 (76.5)	1.80 (0.98, 3.29)
No	309 (79.2)	45 (14.6)	264 (85.4)	1.00
Pain complaint in NRS				
< = 3	121 (31.0)	6 (5.0)	115 (95.0)	1.00
>3	269 (69.0)	58(21.6)	211 (78.4)	5.27 (2.20, 12.58)
Pain complaint in VRS				
None/Mild	147 (37.7)	5 (3.4)	142 (96.6)	1.00
Moderate	144 (36.9)	22 (15.3)	122 (84.7)	5.12 (1.88, 13.93)
Severe	99 (25.4)	37 (37.4)	62 (62.6)	16.9 (6.36, 45.18)

### Magnitude of depression and depressive symptoms

**Fig 1:**
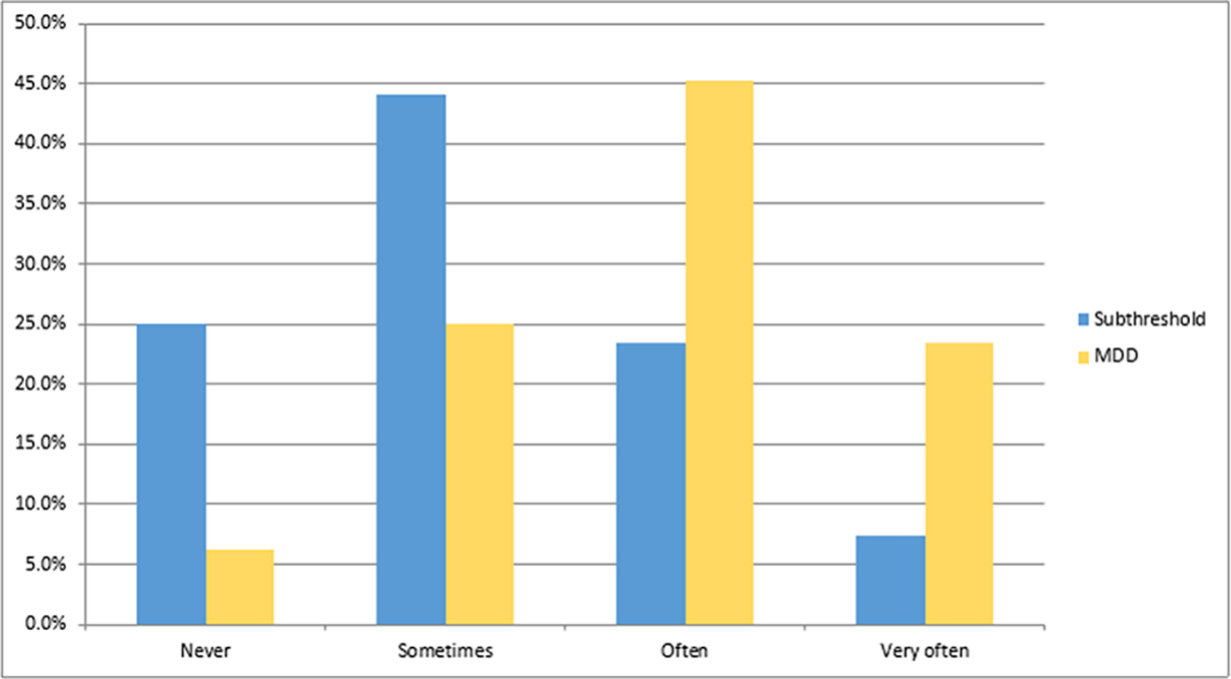
Functional impairment among the DSM related criterion depressed adult cancer patients.

According to DSM related criterion diagnosis, the prevalence of major depression was 16.4% (95%CI: 13.1%, 20.4%) and that of subthreshold depression was17.4% (95%CI: 14.0%, 21.5%). Level of functional impairment among patients with subthreshold depression was relatively low compared to patients with major depression (Fig 1).

**Fig 2:**
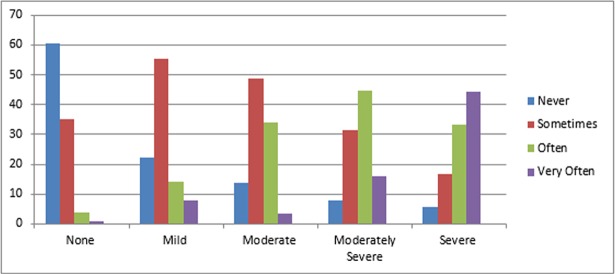
Functional impairment among the PHQ-9 score cut off depression subtypes on adult cancer patients.

Using PHQ-9 score cut off of 5 for mild, 10 for moderate, 15 for moderately severe, and 20 for severe depression, the prevalence of depression was 28.7%, 22.5%, 9.8% and 4.6% respectively. Functional impairment among these groups shows an increasing trend in relation to the severity of the depression (Fig 2).

**Fig 3:**
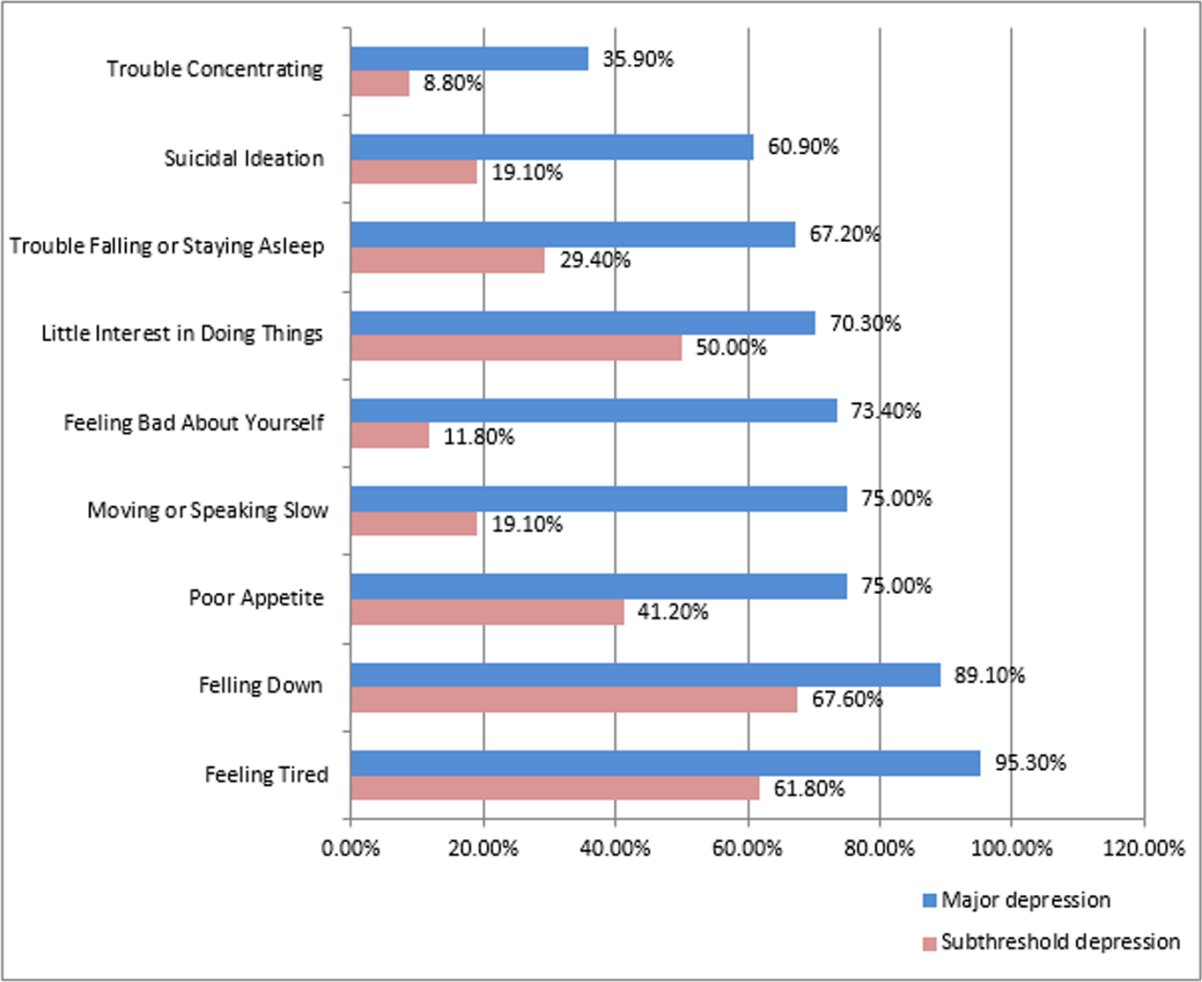
Proportions of depressive symptoms among the depression subtypes.

Among those with major depression and subthreshold depression, feeling tired and feeling down were the commonest symptoms occurring respectively in 95.3% and 89.1% in those with major depression and 61.8% and 67.6% in those with subthreshold depression. Suicidal ideation was observed in 60.9% of those with major depression and 19.1% of those with subthreshold depression (Fig 3).

### Sensitivity analysis

The prevalence of MDD using the score based cutoff diagnosis was more than twice that of the criterion base (DSM IV) diagnosis (39.9%) but all participants who were diagnosed with MDD as per the criterion diagnosis were also diagnosed with depression in the score based system. In the regression model, all significantly associated factors in the criterion based diagnosis, remained significant except social support. Moreover, three additional factors (lack of regular source of income, being in follow up and poor satisfaction with care) were also associated significantly with the score based MDD. (S1 Table).

### Magnitude of pain complaint

As seen in Table 4; the prevalence of pain complaint measured with VRS was 62.3% (95% CI; 57.4, 62.3) and 69.0% (95% CI; 64.3, 73.4) measured with NRS (a rating of > 3, which represents impairing pain). In VRS assessment, 36.9% (95% CI; 32.2, 41.8) rated their pain within the past two weeks as moderate and 25.4% (95% CI; 21.3, 29.9) rated as severe. Again, measured with NRS, 26.2% (95% CI; 22.0, 30.7) of the participants rated the pain they encountered within the last two weeks as 10 which is the most severe pain imaginable. Among the overall participants, 48.5% (95% CI; 43.5, 53.4) were receiving pain medication, which is almost 3/4^th^ of participants with the complaints.

**Table 4:** Prevalence of depression, pain and related factors among study participants (n = 390).

Clinical characteristics (n = 390)	Number (%) Positive	95%CI
Depression		
Major depression	64 (16.4)	13.1, 20.4
Subthreshold depression	68 (17.4)	14.0, 21.5
Suicidal ideation	72 (18.5)	9.1, 21.2
Functional Impairment	268(68.7)	64.0, 73.2
Pain complaint in the last 2 weeks		
As measured by VRS		
No	89 (22.8)	18.9, 27.2
Mild	58(14.9)	11.6, 18.7
Moderate	144(36.9)	32.2, 41.8
Severe	99(25.4)	21.3, 29.9
As measured by NRS		
NRS >3	269(69.0)	64.3, 73.4
Under pain medication(n = 390)	189(48.5)	43.5, 53.4

VRS = Verbal Rating Scale

NRS = Numeral Rating Scale

### Factors associated with depressive symptoms

As briefed in Table 5; in a fully adjusted model, perceived social support and belief about cancer curability and occurrence of pain were significantly associated with the likelihood of having major depression. Those with a perception of poor social support were more likely to have a major depression [Adjusted OR (95%CI) = 2.46(1, 21, 5.00)]. Similarly, those who don’t believe that cancer can be cured were more likely to have a major depression [Adjusted OR (95%CI) = 2.48(1, 30, 4.70)].

Measured with VRS, severe pain [Adjusted OR (95%CI) = 13.17 (4.80, 36.10)] and moderate pain [Adjusted OR (95%CI) = 4.71(1.70, 13.09)] were significantly associated with increased likelihood of having major depression. Similarly, odds of having a major depression was more than four times higher among those with pain score of more than three in the NRS.

**Table 5:** Fully adjusted effects of selected socio-demographic and clinical characteristics on the odds of being depressed.

Patient characteristics	Crude OR (95% CI)	Adjusted OR (95% CI)
Educational status		
No formal education	1.62(0.67, 3.95)	1.30 (0.40, 4.19)
Primary Education (Grade 1–8)	2.45 (1.00, 5.98)	2.58 (0.80, 8.36)
Secondary Education (Grade 9–12)	1.58(0.65, 3.81)	1.47 (0.51, 4.29)
Higher Education (Anything above grade 12 e.g. Diploma, Degree,. . . .)	1.00	1.00
Regular source of income		
Yes	1.00	1.00
No	2.07 (1.25, 3.80)	1.59 (0.72, 3.52)
Perceived Social support		
Good	1.00	1.00
Poor	2.45 (1.34, 4.50)	2.46 (1.21, 5.00)
Health satisfaction		
Poor	2.56 (1.39, 4.69)	1.50 (0.76, 2.95)
Good	1.00	1.00
Belief that cancer can be Cured		
Yes	1.00	1.00
No	2.77 (1.59, 4.82)	2.48 (1.30, 4.70)
Pain complaint in NRS**		
< = 3	1.00	
>3	5.27 (2.20, 12.58)	4.49 (1.84, 11.00)
Pain complaint in VRS^£^		
None/Mild	1.00	1.00
Moderate	5.12 (1.88, 13.93)	4.71 (1.70, 13.09)
Severe	16.9 (6.36, 45.18)	13.17 (4.80, 36.10)

** All effects reported in the table are adjusted for each other and also for pain complain in VRS.

£ = The effect of pain complaint in VRS is not adjusted for pain complaint in NRS because of multi-coliniarity but it is adjusted for all variables indicated in the table.

## Discussion

This study finds a high prevalence of uncontrolled pain, which was also associated with symptoms of major depression. Almost 70% of those with depression had pain complaints.

The prevalence of depression in patients with cancer in this tertiary center is higher when compared to the prevalence of the general population in Ethiopia. The limited studies available from Ethiopia are population (community) based and report a prevalence ranging from 1.2% to 12% [22, 39–43]. The only study conducted in one of the tertiary hospitals in Addis Ababa among people attending the hospital service reported a prevalence of 12.6% [31]. The prevalence in our report was higher than that reported in this clinical sample. Studies in Ethiopia have used different instruments to establish the period prevalence of depression: Self-Reporting Questionnaire [42, 43], Composite International Diagnostic Interview[22, 39–41] and the Patient Health Questionnaire[31]. Comparing the prevalence of co-morbid depression in patients with cancer in our sample against the broader worldwide report, including those of low and middle income countries, the prevalence figure reported in our study falls within the range of the worldwide report albeit at the lower margins. However, functional impairment as a result of depressive symptoms was higher in our study compared with what was found in both high and middle income countries. In the later settings, the prevalence of co-morbid depression ranged from 10% to 58% [15, 16, 18, 25, 28, 44–47]. Rate of depression among cancer patients [46, 48] was higher in studies from the Middle East, which appears to be a reflection of the overall higher prevalence of depression in these countries [21]. Nevertheless, systematic reviews have been consistent with our finding [44, 49].

Studies from Asia and Africa are few in number; however, they have reported higher prevalence. Thus studies from Pakistan and Nigeria reported a prevalence of 61.6% [16] and 37.2% [25]. Again the higher rate in patients from Pakistan might have been a reflection of the generally higher rate of depression in the setting [50]. The study from Nigeria was based on inpatients with more severe illness and pain, which may be the reason for the higher prevalence.

Unmanaged pain was high in our study population but these values fall within the reported prevalence of pain among patients taking anticancer therapy worldwide. A systematic review, mostly based on studies from high income countries, found a prevalence of 59% [51]; similarly a study from Netherlands reported a 44% prevalence [24]. Figures from high and upper middle income countries report lower prevalence, which might be due to a better pain management and an earlier diagnosis and treatment. On the other hand studies from low income countries report higher prevalence. For example, a study from Nigeria reported a prevalence of 87.1%[25]of moderate and severe pain. Although this high figure might relate to the nature of the sample, the higher rate of depression in the Nigerian sample compared to ours might also be relevant.

Consistent with previous literature, this study shows the strong association between depression and cancer pain where the odds of having a major depression is more than four times higher in those with pain complaints. In using a VRS the odds of having a major depression shows an increasing trend towards severity of pain. Different studies in different setups have shown association between depression and pain, prevalence of pain was observed to be much higher together with a depressive symptom than one condition alone[27, 52–54]. Such strong associations were described in both low income [25],high and middle income settings[47, 55]. Studies suggest that pain is a common symptom of depression and again the presence of pain can negatively affect the recognition and treatment of depression making their coexistence a vicious cycle. In some cases pain can be considered as a sign of severity of a disease so patients with pain might think that the situation is getting worse and loose hope, increasing the number of depressed patients, or in other cases depression can sensitizes patients to feel pain and this might exacerbate the number of depressed patients in those with pain complaint. Functional impairment as the result of the depressive symptoms was higher in our study compared with what was found in both high and middle income countries like the report from Indiana (43%)[55], Iran (19.7%)[47] and even the Nigerian study (44%)[25]. Our study participants were either in post operational stage or in advanced cancer stage (III, IV or V).

There are several limitations that should be considered. The study is cross-sectional and direction of association between pain and cancer could not be established. The study was conducted in the capital city in a tertiary hospital. Although this is the only hospital that provides radiotherapy and comprehensive cancer care, the participants are more likely to have more severe illness and also to have more favorable socio-economic status. Therefore, the findings may not be generalizable to the broader population of patients with cancer. Some of the somatic symptoms, which are part of the PHQ9 symptom list, may have been due to the cancer itself or its treatment rather than due to depression. The impact of treatment and pain relief was not assessed in this study. We have also used a criterion based diagnosis for clinical utility reasons than the typical score based diagnosis of depression employed in many studies. The sensitivity analysis confirmed our assumption that the criterion based diagnosis may be a more conservative estimate of the prevalence of MDD and that both categories may be qualitatively similar. This is the first study of its kind in Ethiopia and one of the very few in Africa. Therefore, the study is an important contribution to our knowledge regarding the potential role of depression and pain in the management of cancer in these settings.

## Conclusion

This study reveals that a substantial number of adult cancer patients were depressed and have a suicidal ideation, causing a significant functional impairment. This study clearly demonstrated a significant association between pain complaint and depression among adult cancer patients. Therefore psychological and physical pain assessment should be incorporated as part of standard cancer care.

## Supporting information

S1 Table Fully adjusted effects of selected socio-demographic and clinical characteristics on the odds of being depressed. (DOCX)
