# Supplementary material for: A descriptive analysis of depression and pain complaints among patients with cancer in a low income country
Source: PLoS One. 2018 Mar 7;13(3):e0193713. doi: 10.1371/journal.pone.0193713 (PMC5841758; doi:10.1371/journal.pone.0193713)
Supplement: S1 Table — (DOCX) [file pone.0193713.s001.docx]

**S1 Table: Fully adjusted effects of selected socio-demographic and clinical characteristics on the odds of being depressed**

| **Patient characteristics** | **Crude**  **OR (95% CI)** | **Adjusted**  **OR (95% CI)** |
| --- | --- | --- |
| Marital status |  |  |
| Single | 1.00 | 1.00 |
| Married | 1.25(0.70, 2.24) | 1.95(0.95, 4.00) |
| Formerly Married | 1.89(1.15, 3.09) | 1.69(0.92, 3.10) |
|  |  |  |
| Regular source of income |  |  |
| Yes | 1.00 | 1.00 |
| No | 1.78(1.15, 2.75) | 2.03(1.18, 3.52)* |
| Patient category |  |  |
| New | 1.00 | 1.00 |
| Follow up | 2.07(1.27,3.38) | 1.96(1.09, 3.54)* |
| \| Medication for cancer \| \| --- \| \| Radiotherapy \| \| Radiotherapy +chemotherapy \| \| `Chemotherapy \| |  |  |
|  | 1.95(1.21, 3.14) | 1.61(0.90, 2.88) |
|  | 1.27(0.72, 2.22)z | 1.39(0.70, 2.77) |
|  | 1.00 | 1.00 |
| Health satisfaction |  |  |
| Poor | 2.73(1.75, 4.25) | 1.89(1.11, 3.21)* |
| Good | 1.00 | 1.00 |
| Belief that cancer can be Cured |  |  |
| Yes | 1.00 | 1.00 |
| No | 6.02(3.84, 9.46) | 5.67(3.38, 9.54)* |
| Presence of Other chronic Illness |  |  |
| Yes | 2.19(1.33, 3.59) | 1.77(0.97, 3.25) |
| No | 1.00 |  |
| Pain complaint in NRS** |  |  |
| <=3 | 1.00 | 1.00 |
| >3 | 3.48(2.08, 5.82) | 3.26(1.77, 5.99)* |
| Pain complaint in VRS^£^ |  |  |
| None/Mild | 1.00 | 1.00 |
| Moderate | 2.55(1.50, 4.34) | 2.09 (1.12, 3.89)* |
| Severe | 7.12(3.99, 12.71) | 5.52 (2.81, 10.84)* |

** All effects reported in the table are adjusted for each other and also for pain complain inVRS.

# £ = The effect of pain complaint in VRS is not adjusted for pain complaint in NRS because of multi-coliniarity but it is adjusted for all variables indicated in the table.
